# Brain network analysis for the discrimination of dementia disorders using electrophysiology signals: A systematic review

**DOI:** 10.3389/fnagi.2023.1039496

**Published:** 2023-03-03

**Authors:** Abdulyekeen T. Adebisi, Kalyana C. Veluvolu

**Affiliations:** ^1^School of Electronic and Electrical Engineering, Kyungpook National University, Daegu, Republic of Korea; ^2^School of Electronics Engineering, Kyungpook National University, Daegu, Republic of Korea

**Keywords:** Alzheimer's disease, brain functional network, connectivity analysis, dementia, electroencephalogram (EEG), magnetoencephalogram (MEG), threshold selection

## Abstract

**Background:**

Dementia-related disorders have been an age-long challenge to the research and healthcare communities as their various forms are expressed with similar clinical symptoms. These disorders are usually irreversible at their late onset, hence their lack of validated and approved cure. Since their prodromal stages usually lurk for a long period of time before the expression of noticeable clinical symptoms, a secondary prevention which has to do with treating the early onsets has been suggested as the possible solution. Connectivity analysis of electrophysiology signals has played significant roles in the diagnosis of various dementia disorders through early onset identification.

**Objective:**

With the various applications of electrophysiology signals, the purpose of this study is to systematically review the step-by-step procedures of connectivity analysis frameworks for dementia disorders. This study aims at identifying the methodological issues involved in such frameworks and also suggests approaches to solve such issues.

**Methods:**

In this study, ProQuest, PubMed, IEEE Xplore, Springer Link, and Science Direct databases are employed for exploring the evolution and advancement of connectivity analysis of electrophysiology signals of dementia-related disorders between January 2016 to December 2022. The quality of assessment of the studied articles was done using Cochrane guidelines for the systematic review of diagnostic test accuracy.

**Results:**

Out of a total of 4,638 articles found to have been published on the review scope between January 2016 to December 2022, a total of 51 peer-review articles were identified to completely satisfy the review criteria. An increasing trend of research in this domain is identified within the considered time frame. The ratio of MEG and EEG utilization found within the reviewed articles is 1:8. Most of the reviewed articles employed graph theory metrics for their analysis with clustering coefficient (CC), global efficiency (GE), and characteristic path length (CPL) appearing more frequently compared to other metrics.

**Significance:**

This study provides general insight into how to employ connectivity measures for the analysis of electrophysiology signals of dementia-related disorders in order to better understand their underlying mechanism and their differential diagnosis.

## Introduction

Dementia is the collective name of a group of symptoms that negatively impair memory, thinking, and social capabilities of affected individuals to the extent of interfering with daily life. It is the most researched neurodegenerative disorder and has attracted the attention of experts in various fields of neuroscience. The most prominent and common form of dementia disorder is Alzheimer's disease (AD). Other common forms of dementia include vascular dementia, frontotemporal dementia, and dementia with Lewy body. Although all forms of dementia disorders occur gradually, the effect of the degeneration caused by the diseases is felt almost always at the later stage of life, usually from 60 years of age and above. Unfortunately, while several attempts made to provide a curative treatment for this group of diseases seemingly proved abortive, the global population is getting more skewed toward the young (Liu and McKibbin, 2022), a situation worrisome to the healthcare community. Therefore, it requires more concerted efforts from all facets of the neuroscience research domain to critically understand the underlying mechanisms leading to the development of dementia-related disorders. This is why dementia-based research keeps gaining continuous attention in the research community.

As computation neuroscience forms an integral facet of neuroscience, this field of study has been using connectivity analysis as a tool to understand the brain and its functionalities. Therefore, connectivity defines the physical or statistical connections between regions of the brain or between neuronal populations (Fornito et al., 2019; van den Heuvel and Sporns, 2019). Connectivities are grouped into three types structural, functional, and effective connectivities based on their orientations (Bassett et al., 2018; Park et al., 2018). Structural connectivity entails the physical or anatomical connections among neuronal populations or brain regions. Conversely, both functional and effective connectivities deal with statistical dependencies between physiological time series recorded across different brain regions. While functional connectivity is non-causal, effective connectivity is causal and directional. Generally, neuroimaging and electrophysiology data are used in formulating connectivity-based analysis of dementia-related disorders. Neuroimaging modalities such as structural magnetic resonance imaging (sMRI), functional magnetic resonance imaging (fMRI), diffusion tensor imaging (DTI), and positron emission tomography (PET) have been employed as biomarkers of dementia or as a discriminator of various dementia-related disorders based on connectivity analysis. Electroencephalogram (EEG) and magnetoencephalogram (MEG) are both two common forms of electrophysiology signals which are employed for understanding the mechanisms of dementia and also for the identification of various forms of dementia disorders using functional and effective connectivity frameworks. Although EEG and MEG are not as rich in terms of spatial information as the neuroimaging modalities, they both have a very good temporal resolution. EEG specifically is cheap, portable, and could be easily accessed.

This study focuses on the analysis of electrophysiology signals (MEG and EEG) of dementia-related disorders based on functional ad effective connectivities. The study aims at providing a detailed outline of the updated and overall procedures involved in the diagnosis of dementia-related disorders using MEG- and EEG-based brain network analysis approach. Understanding the current knowledge status about electrophysiology signal applications in computational neuroscience with respect to the identification and discriminatory analysis of dementia-related disorders forms a key goal of this article. To this end, the preferred reporting items for systematic reviews and meta-analysis (PRISMA) are employed to ensure reliable and meaningful study outcomes. The 27 checklist items of the PRISMA protocol, which enable researchers to have accurate and reliable evidence, are strictly adhered in this article (Page et al., 2021a).

In the following sections, the review methodology, results, discussion, and conclusion are presented.

## Methods

### Review standards

The systematic review conducted in this article is based on the preferred reporting items for systematic reviews and meta-analysis (PRISMA) procedures (Page et al., 2021a,b; Sohrabi et al., 2021). The reviewed articles are selected using research questions and research strategy to limit the effect of research expectation and the current review study. The risk of bias was minimized using the Cochrane collaboration method (Cumpston et al., 2019).

### Research questions

- **RQ1**: How could computational approach be used on electrophysiology signals for the understanding of underlying mechanisms of dementia-related disorders?
- **RQ2**: What are the electrophysiological signal-based connectome techniques employed for the understanding of dementia disorders?
- **RQ3**: How could binary networks and weighted brain networks be modeled with the use of electrophysiological signals of dementia-related disorders?
- **RQ4**: How much have electrophysiological signals contributed to the identification or detection of early onset of dementia disorders?
- **RQ5**: What are the graph theory metrics that have been recently instrumental for the analysis of brain functional and effective networks of dementia-related disorders?
- **RQ6**: With regards to the advancement presented in the literature at present, what are the possible research gaps that need to be filled in the aspect of connectivity analysis of electrophysiology signals of dementia-related disorders?

### Study strategy

A comprehensive search of the most recent literature was done independently using the following search engines and databases such as ProQuest, IEEE Xplore, Science Direct, Springer Link, and PubMed from 2016 to 2021. We first search for the following keywords using the following Boolean operators: “functional connectivity” OR “brain functional networks” OR “connectivity” OR “connectome” AND “EEG” OR “Electroencephalogram” OR “MEG” OR “magnetoencephalogram” AND “threshold selection” OR “thresholding” OR “threshold setting” OR “threshold-ing” OR “Binarization” OR “Binary network” OR “Binary matrix” OR “Unweighted network” AND “Dementia” OR “Alzheimer's diseas” OR “AD” OR “Vascular dementia” OR “VD” OR “VaD” OR “mild cognitive impairment” OR “MCI” OR “Frontotemporal dementia” OR “Dementia with Lewy body.”

Due to the word length limitation of some of the search engine used, the combination of keywords used in the previous search terms was further split into the following to accommodate all the employed search engines. Thus, all the groups of keywords mentioned later are searched independently with the presented Boolean operators and the sum of the outputs in all corresponds to the reported search results.

- “Functional connectivity” OR “brain functional networks” OR “connectivity” OR “connectome” AND “EEG” OR “MEG” AND “threshold selection” AND “Dementia” OR “Alzheimer's disease.”
- “Connectivity” OR “connectome” AND “EEG” OR “Electroencephalogram” OR MEG OR “magnetoencephalogram” AND “threshold selection” OR “thresholding” AND “Dementia” OR “Alzheimer's disease.”
- “Connectivity” OR “connectome” AND “EEG” OR “MEG” AND “threshold selection” OR “Unweighted network” AND “Dementia” OR “Alzheimer's disease” OR “Vascular dementia” OR “mild cognitive impairment” OR “MCI” OR “Frontotemporal dementia.”
- “Functional connectivity” OR “brain functional networks” AND “EEG” OR “MEG” AND “threshold selection” OR “thresholding” OR “threshold setting” OR “threshold-ing” OR “Binarization” OR “Binary matrix” OR “Unweighted network” AND “Dementia” OR “Dementia with Lewy body.”
- “Connectivity” OR “connectome” AND “EEG” OR “MEG” AND “threshold selection” OR “Unweighted network” AND “Dementia” OR “Alzheimer's disease” OR “Vascular dementia” OR “mild cognitive impairment” OR “MCI” OR “Frontotemporal dementia.”

### Study selection

**Figure 1:**
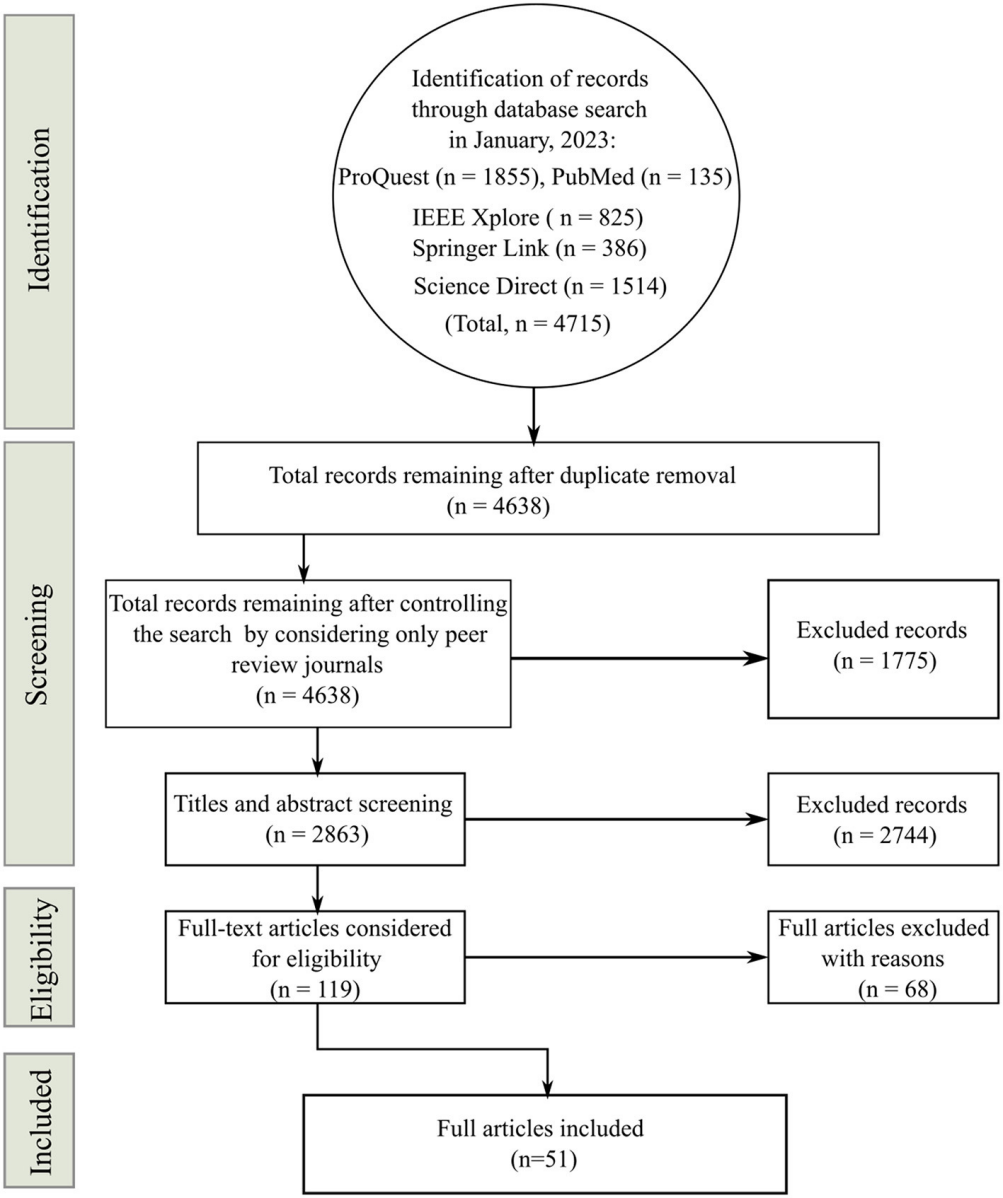
Selection process and the methodology flow chart.

The search produced a total output of 4,715 articles from ProQuest (*n* = 1, 855), PubMed (*n* = 135), IEEE Xplore (*n* = 825), Springer Link (*n* = 386), and Science Direct (*n* = 1, 514). Removal of duplicate articles resulted in a total of 4,638 records. Upon screening the articles considering only peer-review journals and imposition of eligibility criteria, a total of 51 research articles were found to be eligible for the proposed study. The flow chart of the selection process for the study is presented in Figure 1.

### Types of dementia disorders and their electrophysiological signal acquisition

Dementia-related disorders are of various types. The early onset of dementia, i.e., the early but abnormal cognitive impairment state is unanimously known as the mild cognitive impairment (MCI) stage (Petersen, 2004). The later onset of dementia disorders that are known to be irreversible is Alzheimer's disease (AD), which accounts for approximately 70% of known dementia, vascular dementia (VD), which is the second most prominent, dementia with Lewy body (DLB), and frontotemporal dementia, and mixed dementia are the later onset dementia disorders that are known to be irreversible. Although few other disorders are linked to dementia, the scope of this article pays less attention to such. The most common risk factor for these dementia disorders is age. Dementia risk increases with age, especially as one approaches or becomes more than 65 years of age, bearing in mind that dementia is not a part of normal aging. Other suggested risk factors include family history and down syndrome (Livingston et al., 2020).

Differential diagnosis of the later stages of dementia has been very difficult because of their similar clinical symptoms and also overlapping underlying mechanisms. This is one of the reasons for various modalities attempting the development of their differential biomarkers. As electrophysiological signals (EEG and MEG) are among the widely researched modalities for their differential diagnosis, the most recent differential diagnosis of various combinations of dementia is presented in the later part of this article.

#### EEG and MEG signal acquisition protocols for dementia-related disorders

Electroencephalogram system generally comes in two basic forms, clinical EEG devices and consumer EEG devices (Ratti et al., 2017). The former is generally employed in healthcare sector and scientific research setting, while the later is mostly employed in consumer and academic settings. In general, EEG devices comprise electrodes with conducive media, amplifiers with filters, A/D converter, and personal computers. The EEG signals from the scalp surface are captured by the conductive electrode, and the amplifiers bring the captured signals to the range that could be digitized such that the converters express the analog signals in digital form. The digitized signals are stored and displayed on a personal computer. Various brands of EEG equipment are available today for recording both the high-density EEG (with electrodes above 64 channels) or low-density EEG (with less than or equal to 64 electrodes).

Magnetoencephalogram systems, on the other hand, are basically composed of SQUID sensors bathed in a large liquid helium cooling unit, capable of detecting and amplifying the neuronal generated magnetic field on the scalp surface. MEG equipment is housed in a magnetically shielded room to get rid of interference.

The recording protocols for EEG of dementia-related subjects (subjects belonging to MCI, AD, VD, FTD, and DLB) are predominantly resting state data in eyes-open and eyes-closed conditions. This has been the common practice as found in the literature due to the difficulties associated with dementia-related subjects with other cognitive task-based protocols.

### Brain regions and brain waves

Human brain is made up of the cerebrum, the cerebellum, the brain stem, and other components that work concertedly to coordinate the functionalities of the human body (Carass et al., 2011). The cerebrum is the largest and the most prominent part of the brain. It contains the cerebral cortex of the right and left hemispheres of the brain. The cerebral cortex is the outermost layer of the brain which is divided into four different lobes named the frontal, temporal, parietal, and occipital lobes (Rosdahl and Kowalski, 2008). The parietal lobe takes care of the movement, stimuli perception, and recognition. The frontal lobe is mostly responsible for reasoning, movement, emotion, planning, and problem-solving. The temporal lobe takes the responsibility for memory, auditory stimuli, and speech recognition. The occipital lobe coordinates actions related to visual stimuli. However, the combination of functionally integrated and segregated processes is brought about by information transfer among the different brain regions (Churchill et al., 2016).

At the network level within the brain, synchronization of the electrical activities of the neuronal population of different cortical regions leads to the production of brain waves. These waves are generally classified into five broad categories. However, other varieties of classifications are also available in previous studies (Teplan, 2002; Al-Kadi et al., 2013). The most generally acceptable naming of the brain waves is the one that relies on the five frequency bands of the brain waves as: delta (0.5–4 Hz), theta (4–8 Hz), alpha (8–13 Hz), beta (13–30 Hz), and gamma (>30 Hz) (Teplan, 2002; Kumar and Bhuvaneswari, 2012; Jackson and Bolger, 2014). The detail taxonomy, frequency range, description, and other referred properties are shown in Table 1.

**Table 1:** Classification of brain waves and their description (Kumar and Bhuvaneswari, 2012; Ismail et al., 2016; Beppi et al., 2021).

**Frequency waves**	**Frequency range (Hz)**	**Wave description**	**Physiological characters**	**Associated locations**
Delta, δ	0.5–4.0	The slowest brain wave	Highly dominant during sleeping	Frontal region (adults)
		The highest amplitude		Posterior region (children)
Theta, θ	4.0–8.0	Slow brain wave	Dominant during meditation, deep relaxation and dreaming	Thalamic region
		Abnormal in adults and normal in children		
Alpha, α	8.0–13.0	Fast brain wave	Dominant in wakeful but relaxed (eyes closed)	Posterior region
		Found in all ages		
		Depicts white matter	Disappear during open eyes	
Beta, β	13.0–30.0	Faster brain wave	Dominant in attention, anxiety, concentration alertness, thinking and calculation	Parietal and frontal regions
			Associated with behavioral tasks such as problem-solving, decision making and task management	
Gamma, γ	>30	The fastest brain wave	Dominant during high level cognitive tasks	Somatosensory cortex
			Related to perception, language processing and learning	

### Connectivity analysis

Connectivity is a term generally used to depict the concepts for understanding and describing complex systems which frequently occurs in various fields such as neuroscience, geomorphology, system biology, ecology, and social network science among others (Turnbull et al., 2018). Connectivity is the extent of the connectedness of entities within a system. Indeed, connectivity has brought about a scientific transformation in understanding and describing what is perceived as complex systems. Specifically, the brain is considered one of the most complex systems in nature, and hence, connectivity has been one of the most instrumental tools for both the understanding of the structural relationships of the brain components and for mapping its functionalities to various regions. Thus, the brain as a system in neuroscience has been understood and described as composed of neurons, cortical areas, or cortical regions which are the entities whose relationships are defined by connectivity.

There are broadly three types of connectivity in neuroscience and in any other fields of study where the connectivity concept is applicable. These are structural connectivity, functional connectivity, and effective connectivity. Structural connectivity (SC) measures the level of configuration or arrangement of the network. This implies that SC quantifies the actual physical connections between the entities of the network. Functional connectivity (FC) describes the statistical relationship or connectedness between the network entities. Effective connectivity is the influence of one network entity (especially neuron at either the synaptic level or population level, in neuroscience) over another (Friston, 2011) or it could be stated in brevity as the causal relationship between network entities.

Connectivity has been a very important tool unfolding a lot of concepts in brain network science and computational neuroscience for a couple of decades. Several studies with applications in the brain computer interface (BCI), emotion recognition, and identification of brain disorders have employed connectivity analysis as the concept looks promising and remains one of the best choices for neuroimaging and electrophysiology data analysis. Importantly, functional and effective connectivities have found wide applications in electrophysiological data analysis since they both rely on activities of the brain over time series. Thus, EEG and MEG with a very high temporal resolution reflect the optimal neural and dynamic responses in such analysis. Considering the field of brain network science, FC and EC measure the respective statistical and causal relationship between pairs of cortical or scalp regions. These measurements are classified as information-based, linear, or non-linear techniques. They are sometimes classified as time-domain or frequency-domain analysis, and in fact, there are several other criteria for their distinctions. Table 2 shows the overview of the most established methods of FC/EC that are pervasively employed for dementia-related disorders analysis using electrophysiology signals, within the last 5 years.

**Table 2:** List of functional and effective connectivity techniques used recently for dementia-related disorder analysis of electrophysiology signals within the last 5 years and their respective properties.

**Estimator**	**Variable**	**Domain**	**Signal component**	**Linearity**	**Volume conduction**
Phase Lag Index (PLI)	Univariate	Frequency	Phase	Non-linear	Less Sensitive
Weighted phase lag index (wPLI)	Univariate	Frequency	Phase	Non-linear	Less sensitive
Partial Coherence (PC)	Multivariate	Frequency	Phase	Non-linear	Robust
Coherence (Coh)	Multivariate	Frequency	Phase	Linear	Highly sensitive
imaginary part of Coherence (iCoh)	Multivariate	Frequency	Phase	Linear	Highly sensitive
magnitude square Coherence (msCoh)	Multivariate	Frequency	Phase	Linear	Highly sensitive
Phase order parameter (POP)	Univariate	Frequency	Phase	Non-linear	Highly sensitive
Synchronization likelihood (SL)	Univariate			Non-linear	Sensitive
Phase coupling estimation (PCE)	Multivariate	Frequency	Phase		
Lagged linear connectivity (LLC)	Multivariate	Frequency	Amplitude and phase	Linear	
Phase transfer entropy (PTE)	Univariate	Frequency	Phase	Non-linear	Less sensitive
Normalized phase transfer entropy (dPTE)	Univariate	Frequency	Phase	Non-linear	Less sensitive
Permutation disalignment entropy (PDI)	Univariate	Time	Phase	Non-linear	Robust
Lagged phase synchronization (LPS)					
Correlation coefficient (CC)	Univariate	Time		Linear	Highly sensitive
Directed transfer function (DTF)	Multivariate	Frequency		Linear	Sensitive
Amplitude envelope correlation with correction (AEC-c)		Frequency	Amplitude	Linear	
Phase locking value (PLV)	Univariate	Frequency	Phase	Non-linear	Highly sensitive
Phase synchronization index (PSI)	Univariate	Frequency	Phase	Non-linear	Highly sensitive
Phase coherence (PC)	Univariate	Frequency	Phase	Non-linear	Highly sensitive
Lagged coherence (LC)					
Granger causality (GC)	Multivariate	Time and frequency		Linear	Less sensitive
Mutual information (MI)	Univariate	Time		Linear	Robust
Normalized mutual information (dMI)	Univariate	Time		Linear	Robust
Epoch based entropy measure (EpEn)	Univariate	Time		Linear	Robust
Generalized composite multiscale entropy vector (GCMSEV)	Univariate	Time		Non-linear	Less sensitive
Complex tensor factorization	Univariate	Time		Non-linear	
Orthogonal least square (ROLS)	Univariate	Time		Non-linear	
Permutation jaccard distance (PJD)	Univariate	Time		Non-linear	
imaginary phase locking value (iPLV)	Univariate	Frequency	Phase	Non-linear	Highly sensitive

**Figure 2:**
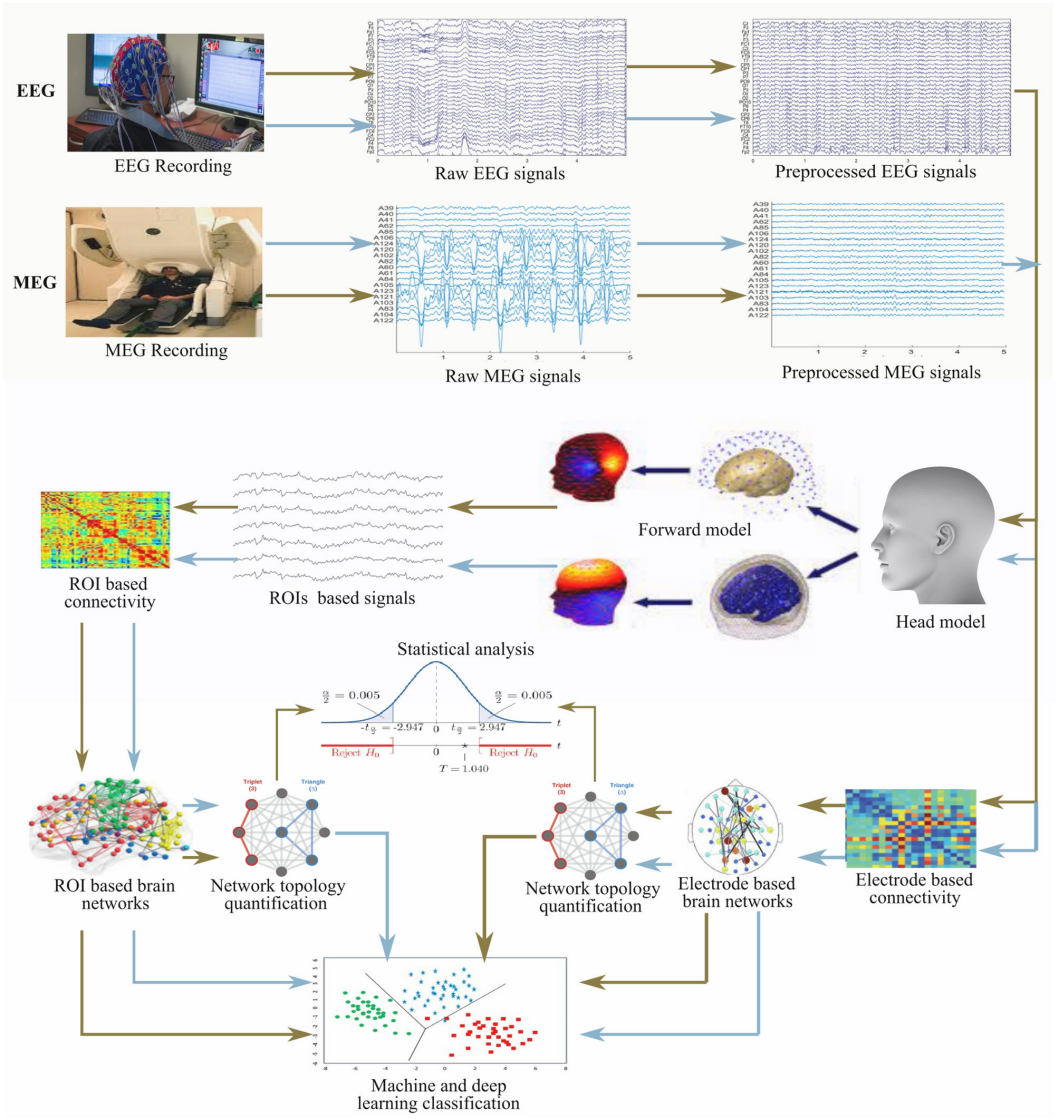
General framework for the construction and analysis of brain network from electrophysiology signals (EEG and MEG).

The properties of the connectivity measures listed in Table 2 are not similar and thus, they have their associated merit(s) and demerit(s). It is, however, unfortunate that there is no golden rule for selecting optimal technique, and so it is quite challenging to select a connectivity measure for a particular framework. A general rule of thumb is to employ a univariate analysis technique when the feature of a single signal from a neurophysiological state is of interest and also when too much care about the sensitivity of cognitive states within the individual is not required. Moreover, multivariate analysis techniques are usually employed when different neurophysiological states are jointly treated in an analysis. Linear connectivity measures are applied when little or no tolerance for noise is desired whereas nonlinear measures are generally used for non-linearity detection in brain activity. Analyses involving the phase component of the signal is considered more sensitive to the detection of brain state compared to connectivity analysis involving the use of amplitude component. The general framework detail of the construction and analysis of brain networks from EEG and MEG is presented in Figure 2.

### Influence of EEG and MEG channel densities on connectivity analysis

One of the choices that have to be made during the consideration of the analysis pipeline of brain functional connectivity based on electrophysiology signals is the choice of the density of the modality (EEG/MEG). Classically, EEG is considered to have an excellent temporal resolution among the various brain imaging modalities. Its associated poor spatial resolution has always been a point of concern. In order to have an improvement on the spatial resolution of EEG, high-density EEG (HD-EEG) montage which employs higher spatial sampling of the scalp electrodes than the standard 10–20 low-density (LD-EEG) montage has been adopted. The minimum number of EEG electrodes required for HD-EEG montage is typically 64 channels (Seeck et al., 2017), and usually, as more electrodes are added to this number, there is an incremental, but diminishing spatial resolution (Sohrabpour et al., 2015). However, the use of HD-EEG has been limited in many applications including those of dementia-related disorders due to the equipment acquisition cost, experimental setup, analysis and interpretation time (Chu, 2015).

Unlike the case of EEG, the number of MEG sensors has not been a very serious concern in various applications. While EEG sensors are placed directly on the scalp surface, MEG sensors are positioned around the subject's head rather than touching the scalp (Singh, 2014). However, it has been shown that the closer the proximity of MEG sensors are to the brain, the better the spatial resolution and the neuronal current information provided by the modality (Boto et al., 2016). In this respect, the number of EEG and MEG sensors employed in the included articles has been identified.

### Influence of epoch length on the connectivity analysis of electrophysiology signals

Another methodological consideration that may bias the topology of functional networks based on EEG and MEG signals is the choice of epoch length. Usually, the epoch length of EEG signals for functional connectivity analysis ranges from 1 s to several seconds. The inconsistency associated with the selection of epoch lengths often impedes objective comparison between the outcomes of various studies. Among the various uncertainties that have to do with the epoch length of EEG and MEG is that the choice of epoch length required for optimum results in that the analysis of connectivity measures is not the same. For instance, in Fraschini et al. (2016), the effect of epoch length on estimated EEG functional connectivity and brain network organization was investigated using PLI and AEC. Therein, a decrease in functional connectivity was found as the epoch length increased and with the attainment of stability at 12 s and 6 s for PLI and AEC, respectively. In order to recognize the common choice of epoch length employed in the analysis of functional connectivity of dementia-related disorders, the various epoch lengths employed in all the articles included in the study are identified.

### Graph theory approach

Graph theory application to neurophysiological (especially electrophysiological data) in the area of computational neuroscience has gained a lot of attention over the last two decades. This field of study which is of Mathematics origin has been employed in the area of neurological disorder diagnosis, affective computing, and brain-computer interface. However, our focus here is to highlight its recent usage in computational cognitive neuroscience and specifically in the diagnosis of dementia-related disorders.

**Figure 3:**
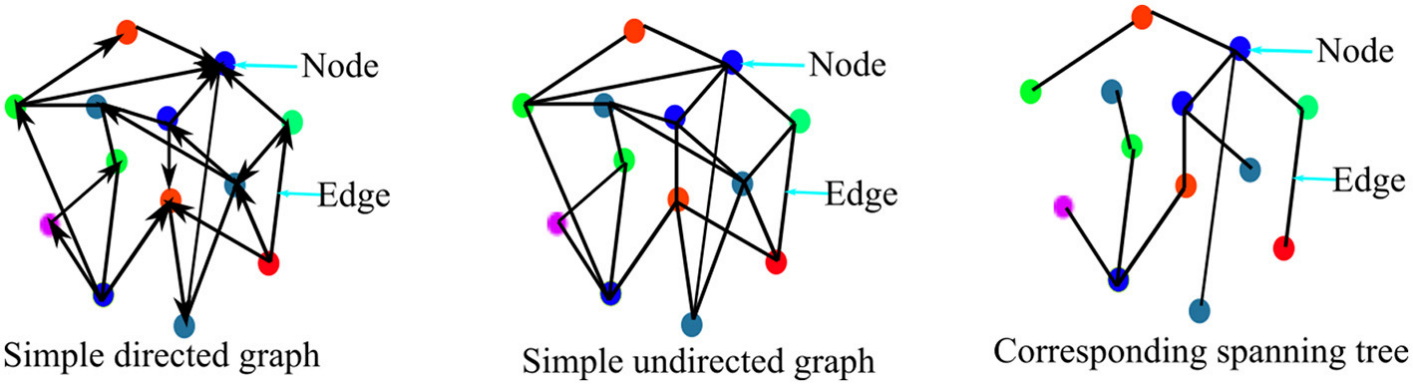
Representation of the simple graph containing 13 nodes and 19 edges.

In general, graph theory presents data as a graph comprising of a set of nodes or vertices (*V*) connected together by edges set (*E*). Hence, a graph *G* is defined as *G*(*V, E, A*), such that *A* is the adjacency matrix which is the matrix that describes the relationship between the set of vertices *V* in the graph. Therefore, studying human brain networks involves representing the brain regions (which could be cortical areas or EEG/MEG sensors location on the scalp) as nodes and the relationship (be it physical, statistical, or causal) between the set of nodes as the edges. A graph can either be weighted or unweighted. A weighted graph is one in which the edges are assigned weights such that the weight of an edge corresponds to its size. Conversely, an unweighted or binary graph is one in which an edge either exists or does not exist between two nodes such that an edge exists if its value is “1” and does not exist if its value is “0”. A graph could be directed or non-directed. A directed graph is one in which the relationship between two nodes is one way. The non-directed graph is one in which the relationship between two nodes is bidirectional. An example of simple binary directed and non-directed graphs is presented in Figure 3 for illustration purposes.

### Electrophysiology signal-based functional brain network construction and analysis framework

The framework for the construction and analysis of the brain functional networks of electrophysiological signals of dementia-related disorders involves the procedures that will be described in the following sections. Special emphasis would be laid to the most recent approaches used in the literature within the survey frame. The objective here is to itemize the procedures in a way that would be useful to the non-expert and semi-expert researchers in the field.

#### Nodes/vertices definition

The first procedure in formulating the brain functional network from electrophysiological and other neurophysiological data generally is to specify the network nodes/vertices. In brain networks, the nodes are the cortical regions or the sensors' locations on the surface of the scalp. Results and analysis of brain functional networks largely depend on the way the network nodes are defined. There are two different approaches to the definition of nodes in EEG and MEG data: The first approach is the use of sensor/channel or channel names. This approach depends on the standard definition of the EEG/MEG sensors placement, and this approach is commonly employed in EEG-based brain functional network construction and analysis. The drawback of this approach is the issue associated with volume conduction which reduces the spatial resolution of the data. The second approach is the source reconstruction approach. This approach relies on the standard definition of the brain region of interest (using a standard brain atlas). It involves the computation of the source space by solving the inverse problem after pre-processing and segmenting the signals into epochs such that the electrode locations in 3D are decided using the software acquisition system. Two main procedures are involved in solving the inverse problem, viz: (i) identification of the head model to be used (with special reference for the realistic head model) and (ii) source localization evaluation in the head model for dipole source location identification. This would then be followed by the reconstruction of the time course. Various algorithms developed for the purpose of source reconstruction have been implemented in free software such as EEGLAB, Brainstorm, Fieldtrip, sLORETA, and MNE.

In general, it is always a requirement to record high-quality signals from the scalp surface for effective analysis. Specifically, subjects with dementia-related conditions are a bit difficult to monitor, so investigators must be extra meticulous in the process of data acquisition or recording in order to achieve very accurate signals. Upon deciding the kind of node definition approach, electrophysiological data must be filtered, denoised, and also separated from various contaminating artifacts such as eye blinks and muscle artifacts. EEGLAB, Brainstorm, and Fieldtrip. are the common freely available software for electrophysiological data pre-processing.

#### Edges definition

Edges definition is a very critical step in brain functional network construction and analysis. Edges in brain networks represent the connections between pairs of cortical areas or scalp sensor locations. In the network construction process, the network edges are formulated through the use of structural, functional, and effective connectivity metrics depending on the type of network in question. For the fact that we are less interested in the structural network in this article, the focus will be on edges definition with respect to functional and effective networks which have been discussed in the previous section. Upon the selection and implementation of appropriate connectivity measures, the edges are either directed or non-directed. Given a multi-channel EEG/MEG, the application of connectivity measure results in the formation of a connectivity matrix, *A*. If the number of node specified in a network is *N*, the connectivity matrix is usually *N*×*N* symmetric matrix where the rows and columns of the matrix represent the node numbers, i.e., *A*_*ij*_ denotes the statistical relation between channels *i* and *j* or region *i* and *j*.

Next, in edge definition, the connectivity matrix obtained directly from the connectivity measure is converted to graph adjacency depending on the type of network. Although the use of the unweighted network is very popular in the construction and analysis of dementia-related brain functional networks, it is not free from the methodological issue which opens room for the consideration of weighted and other form of brain functional sub-networks such as minimum spanning tree (MST) networks and minimum connected component (MCC) networks. Formulating unweighted brain functional network usually involves the determination of inclusion and exclusion borderline for selecting edges as part of the network by selecting the appropriate threshold. A lot of methods have been proposed for appropriate threshold selection in the literature. These methods could be grouped into two broad categories, viz: the arbitrary and data-driven selections.

- The arbitrary/random value selection: This involves the selection of threshold value by choosing a constant value randomly such that an edge is kept as a true edge if and only if its value is above the randomly selected threshold value. The problem with this approach is that the formulated network density varies from subject to subject and in fact, some subjects have networks that are connected, while others have disconnected networks (Wang J. et al., 2016; Engels et al., 2017; Vecchio et al., 2017; Mammone et al., 2018b; Zhao et al., 2019; Li et al., 2021; Toural et al., 2021). This makes the final comparison of the networks across subjects or groups difficult and may appear biased.
- Data-driven threshold selection: This involves the selection of a threshold by either developing a technique or using a particular criterion based on which the threshold value will be chosen. There are various approaches developed under this broad category and the most frequently and recently used are summarized later.
  1. Sparsity thresholding/Threshold selection based on edge density: This involves the selection of threshold by fixing an edge density, average degree of the network, or by selecting a certain fixed percentage of the total number of possible connections in the fully connected network (Afshari and Jalili, 2016; Wang C. et al., 2016; Jalili, 2017; Mammone et al., 2018a; Song et al., 2019; Mehraram et al., 2020; Youssef et al., 2021). In this way, the actual values of threshold across subjects or groups are not constant. By this, the connectedness of the network is expected to be more preserved compared to the case of random value selection. However, using this approach does not also guarantee the absence of disconnection in the formulated network. Thus, unbias network analysis is also not fully guaranteed using this approach.
  2. Statistical approach to threshold selection: This threshold selection technique involves the selection of threshold across networks using a particular statistic of the total possible connections in the fully connected network. Examples of this threshold selection approach include the keeping of connection within a particular confidence interval (it is common to use a 95% confidence interval) (Bassett et al., 2006), the selection of the median value of the connectivity values as threshold (Yu et al., 2018), and the selection of connections above a particular confidence level. However, this approach is also not free from the question of network comparison bias.
  3. Threshold selection based on maximizing global cost efficiency: This is a thresholding technique that relies on the use of a fundamental property of the network, global efficiency. In this approach, the network cost which is the ratio of the sum of edges in a network to the total number of edges in the fully connected network is computed such that the difference between the global efficiency and the cost value gives the global efficiency cost. Thresholding using this approach involves selecting the arbitrary threshold at which the maximum global cost efficiency occurs for the network (Cai et al., 2020). Similar to other approaches as mentioned earlier, this approach also does not guarantee unbiased comparisons of network measures across a group of subjects. The connectedness of the network is also not automatically guaranteed using this approach.
  4. Threshold selection by keeping the giant component: This threshold selection approach is developed from the technique to keep as many edges so as to maintain connectedness of not less than 99% of the entire nodes in the network (Bassett et al., 2006). Threshold selection keeping giant component is one in which a threshold value is based on percolation such as keeping a minimum threshold that maintains the connectedness of the giant component (Bordier et al., 2017). Although this approach has not been so common, the basic advantage of this method lies in the topological integrity of the original network obtained directly from the connectivity matrix. However, there is no guarantee that spurious networks are eliminated using this approach. This approach looks problematic for network formulation from effective connectivity where the connectivity matrices are mostly sparse.

The earlier threshold selection approaches are commonly used for unweighted/binary brain network formulation despite their associated drawbacks and differences as studied in Jalili (2016). However, because of the methodological issues associated with those techniques, the use of the weighted network has been adopted (Franciotti et al., 2019). Unfortunately, the use of the weighted network does not also avoid the thresholding dichotomy as spurious connectivity values which need to be eliminated are still present. Thus, the use of some of the earlier threshold selection criteria is also employed to remove spurious connections in weighted network analysis (Chen et al., 2019).

Furthermore, it is obvious that the use of weighted network does not perfectly assure freedom of network analysis from the methodological issues associated with threshold selection bias. Therefore, many brain network analysts have employed the use of the minimum spanning tree, MST for non-biased network analysis and comparisons (Jalili, 2016; Yu et al., 2016; López et al., 2017; Das and Puthankattil, 2020; Požar et al., 2020). A spanning tree is an acyclic network component/sub-network where all the network nodes are connected with the minimum possible number of edges. Typically, for an *N*-nodes graph, the spanning tree of the graph connects the N-nodes with (*N* − 1) edges as illustrated in Figure 3. Since a graph usually has many spanning trees depending on the size of the graph, the spanning tree with a minimum cost of wiring is called the minimum spanning tree. In the brain functional network, the maximum spanning tree is usually considered the MST. Hence, MST is perceived to be inherently carrying the original network properties and so could be appropriate for unbiased network analysis and comparison (Tewarie et al., 2015). However, the minimum spanning tree-based network is not without its shortcomings. Spanning trees usually are acyclic in nature and so computation of graph metrics such as clustering coefficient and transitivity involving cycles are not possible using minimum spanning trees. Improving capability and attempting to solve the issues associated with MST, Dimitriadis et al. (2019) proposed the use of orthogonal MST (OMST), in which MST is extracted successively one after the other for as long as a given condition is satisfied.

Owing to the identified issues related to unweighted, weighted, and MST earlier, the minimum connected component (MCC) concept is proposed to objectively produce networks that could be analyzed and compared objectively. MCC is a special kind of spanning subgraph that connects all the nodes present in a graph with the minimum number of maximum weighted edges. For a graph having *N*-nodes, there are at least (*N*−1) edges and at most ($\frac{N\left(N-1\right)}{2}$). In Vijayalakshmi et al. (2015) and Jalili (2016), MCC is proposed and employed for the quantitative measurement of cognitive activity and detection of cognitive impairment. However, MCC may not also be completely free from unbiased network comparison as the network density of MCC for different subjects in a group cannot be‘constant.

#### Graph theory analysis of brain networks

Upon the formulation of unweighted, weighted, MST, or MCC networks, the topologies of the formulated networks are quantified using graph theory measures. With respect to dementia-related disorders, topological quantifications are employed for the discrimination of dementia onsets and various types of dementia disorders at the network level. The fundamental graph theory measures used for the brain network analysis are the measures of functional segregation and integration, respectively. Common measures of functional segregation include the clustering coefficient, transitivity of the network, and local efficiency. Similarly, path length and global efficiency are the common measures of functional integration. Other common metrics have been proposed at various times to access the local and global properties of brain networks, and in fact, various toolkits have been developed to analyze and visualize the network topological properties (Rubinov and Sporns, 2010; Xia et al., 2013; Wang et al., 2015; Mijalkov et al., 2017). The common graph measures that are frequently used for the analysis of dementia-related disorders and their basic definitions are summarized later:

1. Local measures: These are graph measures that are directly computed at the nodal level of a graph. It must be noted here that some measures could be employed at both local and global levels, hence, such measures are only discussed under the heading of global measures.
  - Connection level metric (CLM): This is a graph metric that quantifies the difference in the synchronization of two nodes. It helps to determine the significant connections by testing the significance of the connection strength of any two nodes (Duan et al., 2020).
  - Local efficiency (LE): This is the average efficiency of transferring information among the nearest neighbors of a given node (Jalili, 2017; Cai et al., 2018).
  - Degree (K): This is the total number of links incident to a node/vertex in a network (Li et al., 2019).
  - Node betweenness centrality (NBC): The betweenness of a node is the number of shortest paths passing through a node in a graph. It is a pointer to the influence a node has on the information flow in a graph/network (Jalili, 2016; Abazid et al., 2021).
  - Node strength (NS): Node strength measures the node's contribution to the entire network by taking the sum of the weights of the link and joining it to the adjacent nodes (Hata et al., 2016; Duan et al., 2020).
  - Participation coefficient (PC): This is a measure that quantifies the participation of a node in different network layers according to degree distribution (Cai et al., 2020).
  - Versatility (Vers): This is the measure of how a particular node is closely affiliated with the network community (Duan et al., 2020).

2. Global measures: These are graph measures computed at the graphical rather than the nodal level. The commonly used global graph measures are summarized later;
  - Attack tolerance (ATol)/network resilience: This is a measure of the ability of a network to maintain its local and global efficiency when a certain percentage of its hub nodes are removed/attacked (Afshari and Jalili, 2016; Duan et al., 2020).
  - Assortativity (Ac) : This is the resiliency of a network to undergo random or internal failure/attack (Jalili, 2017). It is also expressed as the ease with which a node links to other nodes of a similar degree.
  - Characteristic path length (CPL): Path length is one of the most important and frequently used graph metrics for measuring functional integration of the network. It is the average of the shortest path length of all the possible nodes in a network (Vecchio et al., 2017; La Foresta et al., 2019). It is inversely proportional to the functional integration of the network.
  - Clustering coefficient (CC): This is one of the most important fundamental graph theory metrics used in brain network analysis to access the functional segregation of the network. It is the measure of the extent to which nodes tend to form a cluster together in a graph (Chen et al., 2019; Duan et al., 2020).
  - Connection density index (CDI): This is the ratio of the total number of links in a graph to the maximum possible number of links in the fully connected graph (Dattola et al., 2021).
  - Eigenratio/synchronizability (EigR): This is the ratio of the largest eigenvalue of a graph to the Fieldler value/algebraic connectivity of the graph. Theoretically, it is the measure of the synchronizability of the network (Jalili, 2017).
  - Global efficiency (GE): This is an important network measure used for the quantification of functional integration, i.e., the efficiency of information transfer (Mammone et al., 2018a; Franciotti et al., 2019). It varies inversely as the average shortest path length of the graph such that the higher it becomes, the faster the transfer of parallel information in the network and the better the integration of information.
  - Graph complexity index (GCI): This is the metric used for measuring the complexity of a network. the GIC varies between “0” and “1” and the larger the value of GIC, the more complex the network becomes (Wang J. et al., 2016; Yu et al., 2018).
  - Modularity index (Mind): Modularity index is a measure of the structure and topology of the network according to edges arrangement statistically (Jalili, 2016).
  - Randic index (Rind): The randic index of a network quantifies the extent of the connectedness of a network. It is inversely proportional to the network connectedness such that the complete graph/network has the minimum randic index (Dattola et al., 2021).
  - Small worldness (SW): Small-worldness is the ratio of the normalized clustering coefficient to the normalized path length of a network. It is an important measure for accessing network functional segregation and it conveys integrated information on global and local network characteristics (López et al., 2017; Cai et al., 2018).
  - Transitivity (Trans): Transitivity is a global measure of the clustering coefficient of a network that is collectively normalized such that it does not suffer unnecessarily from the influence of nodes with low degrees (Jalili, 2017).
  - Vulnerability (Vuln): Vulnerability is the measure of connectivity damage as a result of nodes removal from the network (Wang J. et al., 2016).

3. Minimum spanning tree measures: As pointed earlier, MST network is proposed for brain network analysis to basically avoid methodology bottlenecks associated with threshold selection and also to enable an unbiased network comparison. Since the topological structure of MST is somewhat different from those of the parent network/graph, its properties are measured using unique graph theory metrics. The most frequently and recently used graph measures for MST are summarized later;
  - Node degree (Deg): Node degree in a tree is the number of neighbors adjacent to the node in the given tree. The maximum node degree in an MST is always of special importance (Požar et al., 2020; Youssef et al., 2021).
  - Tree diameter (Diam): The tree diameter is the maximum distance between any two nodes in a tree (Das and Puthankattil, 2020; Youssef et al., 2021).
  - Tree eccentricity (Ecc): The tree eccentricity is the distance of a particular node to the farthest node away from it (Požar et al., 2020; Youssef et al., 2021).
  - Betweenness centrality (BTC): Betweenness centrality, just like the graph betweenness centrality, measures the to which a node is located between the path of two other nodes (Briels et al., 2020b; Youssef et al., 2021). The maximum betweenness centrality of a tree is usually used in characterizing the tree.
  - Leaf fraction (LFrac): This is the ratio of leaf number to the maximal possible leaves in the tree (Das and Puthankattil, 2020; Youssef et al., 2021).
  - Tree hierarchy (Hier): The tree hierarchy is a metric that measures the equilibrium between the reduction of diameter and overload prevention in a tree (Požar et al., 2020; Youssef et al., 2021).
  - Dissimilarity index (Dind): The dissimilarity index is a metric used in measuring the extent of topological difference between two trees. Basically, it quantifies the amount of information required to transform one tree into another (Požar et al., 2020).
  - Survival ratio (SuR): The survival ratio of MST is used to quantify the similarity between MST and a reference by taking the ratio of the common links to the total number of possible links in the MST network (Yu et al., 2016).
  - Mean weight (Wei): This is the average weight of all the links found in a tree. It works in the same way as the connection level metric of a traditional network (Yu et al., 2016).
  - Degree correlation (Dcorr): The degree correlation of a tree measures the tendency of nodes to connect with nodes having comparable/similar degrees. In other words, it measures how similar nodes are in a tree (Yu et al., 2016; López et al., 2017).
  - Tree efficiency (TEff): The tree efficiency indicates how close the tree diameter is to the lowest possible value (Yu et al., 2016).
  - Tree divergence (Div): The tree divergence, also called kappa, is the measure of the broadness of the degree distribution in a tree (Yu et al., 2016; Požar et al., 2020).

#### Dementia-related conditions discrimination

In most cases, the last stage of the connectivity analysis of electrophysiological signals of dementia-related disorders is the stage at which the conditions of the subjects involved in the analysis are discriminated or identified. This is because, the utmost goal of such analysis is to achieve a differential diagnosis of the disorders to aid therapeutic measures development. Two different approaches are previously being considered for achieving this and the two approaches would be looked into in what follows;

1. **Statistical Analysis**: As discussed in the previous section, the formulation of brain networks is almost always followed by network quantification using graph theory metrics. Upon the computation of graph theory measures, statistical methods are applied to compare the between group network properties for possible significant difference identification. Sometimes, the results of the real brain networks obtained from electrophysiological data are statistically compared with the theoretical network such as random, lattice, or scale-free networks. Accessing statistical variability between different groups of dementia-related disorders depends on factors such as the size of the dataset (number of subjects considered or number of samples considered) and the underlying distribution of the samples. As it is the case in most other applications of statistical analysis tools, 95% confidence intervals are commonly employed for between dementia groups' discrimination using parametric statistics such as one-way ANOVA. Non-parametric statistics such as Kruskal–Wallis method (Kruskal and Wallis, 1953; Corder and Foreman, 2011), permutation statistics, and bootstrapping (Moore, 1999) are also widely considered appropriate especially when the data fail to be normally distributed.
2. **Learning Approach**: Machine and deep learning approaches have been consistently and continuously gaining attention in the classification and identification of dementia-related disorders on the basis of brain network framework. Feature extraction techniques are employed to mine useful features before the condition-based classification using appropriate classifiers. Similarly, deep learning approaches such as feed-forward neural networks have been employed for the dementia-related condition-based classification. Table 3 shows the summary of the most recent classification frameworks on electrophysiology-based functional/effective brain networks of dementia-related disorders using learning techniques.

**Table 3:** Recent learning-based classification of dementia disorders using various functional connectivity features.

**Data type**	**Study pathology and population**	**Classifier**	**Highest accuracy(*%*)**	**References**
EEG	AD-32, DLB-25, PDD-21, NC-18	Random forest	66 ± 13	Mehraram et al., 2020
EEG	sMCI-28, pMCI-25 and AD-17, NC-24	Svm with cubic kernel	83.3 and 85.5	Duan et al., 2020
EEG	sMCI-28, pMCI-25 and AD-17, NC-24	RESNET-18	93.42 and 98.54	Duan et al., 2020
EEG	aMCI-28, NC-21	Svm with rbf kernel	66.6 ± 17	Li et al., 2021
EEG	AD-30, NC-30 open eyes and closed eyes	Takagi-Sugeno-Kang network	94.78 and 97.3	Yu et al., 2020
EEG	MCI-7, NC-7	KNN and neuro-fuzzy-kNN	95.9 ± 0.4 and 97.2 ± 0.5	Jamaloo et al., 2020
EEG	AD-20, NC-20	KNN	90 ± 5.34	Zhao et al., 2019
EEG	AD-25, NC-20	Svm with rbf kernel	83	Jalili, 2017
EEG	AD-20, NC-20	Svm (multiplex network features)	92.5	Cai et al., 2020
EEG	aMCI-43, NC-51	Random forest	87.2	Youssef et al., 2021
EEG	AD-14, NC-14	Svm with rbf kernel	98.9	Yu et al., 2018
EEG	MCI-13, NC-27	Linear discriminant analysis	86.5	Požar et al., 2020
EEG	AD-25, NC-26	Linear discriminant analysis	94	Afshari and Jalili, 2016
EEG	AD-15, NC-15	Svm with rbf kernel	96	Song et al., 2019
EEG	ADD-42, DLB-34, PDD-34, NC-40	Linear discriminant analysis	84	Babiloni et al., 2018
EEG	AD-MCI-30, DLB-MCI-23, NC-30	ROC curve	75	Babiloni et al., 2019
MEG	sMCI-27, pMCI-27	Svm with rbf kernel	100	Pusil et al., 2019
EEG	sMCI-76, NC-53	KNN and svm	>95	Xu et al., 2021
EEG	AD-20, MCI-14, NC-53	2 layer feed-forward neural network	>94.44	Toural et al., 2021
EEG	AD-118, MCI-135, NC-198	Penalized logistic regression	>70 − 80	Farina et al., 2020
EEG	AD-28, MCI-28, NC-22	Svm with rbf kernel	>90 − 100	Abazid et al., 2021
EEG	aMCI-139, naMCI-58	Naive Bayes Algorithm	89	Kim et al., 2022
EEG	AD-20, NC-20	Graph neueral network (GNN)	92	Klepl et al., 2022
MEG	SCI-105, MCI-45, AD-127, DLB-27, FTD-33	Graph neueral network (GNN)	Variable	Scheijbeler et al., 2022

## Results

### Study selection

**Figure 4:**
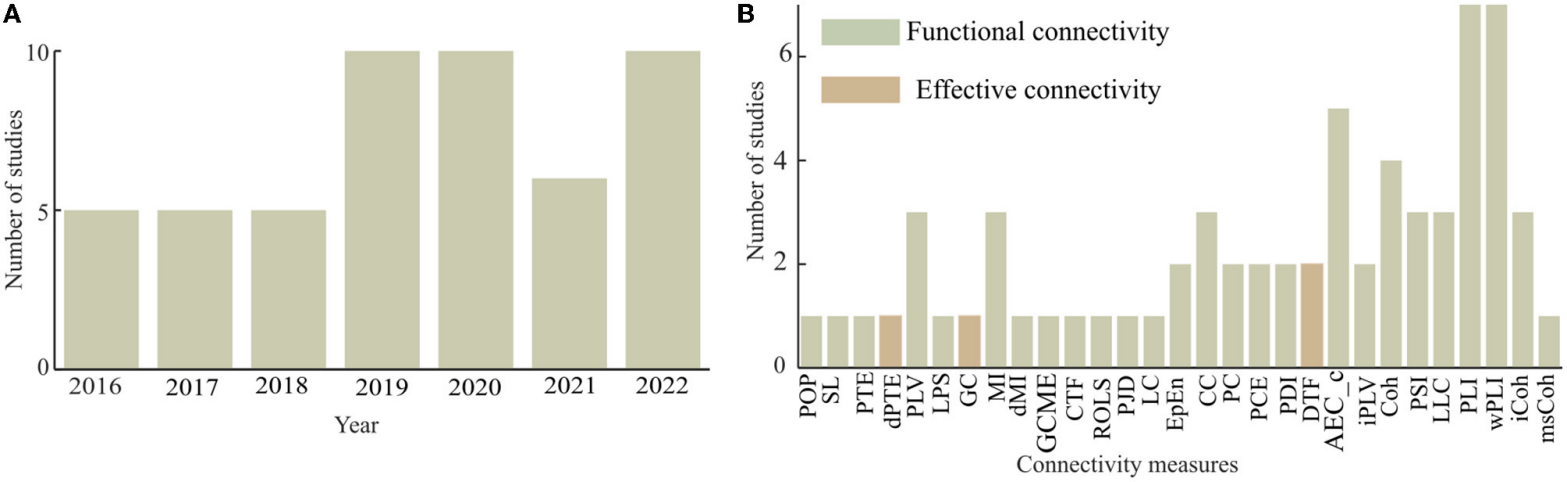
Frequency of the usage of connectivity measures for dementia-related disorders with **(A)** annual distribution of the reviewed studies and **(B)** frequency distribution of the usage of connectivity measures.

Based on the selection criteria, a total of 51 articles were selected among which 25 articles (49%) of the total were published between 2016 and 2019, and the rest (51%) were published within the last 3 years (2020–2022) (see Figure 4A). Hence, the present study demonstrated an increasing trend in the research domain of brain functional network analysis of dementia-related disorders using electrophysiology signals. It is expected that future studies will grow further compared to the present trend.

### Connectivity analysis

This article considered studies that employed both functional and effective connectivity measures as shown in Figure 4B. Out of the total of 29 different connectivity measures found in the considered articles, four articles (about 7.84%) employed effective connectivity measures. The effective connectivity measures considered include phase transfer entropy (PTE), normalized phase transfer entropy (dPTE), granger causality (GC), and directed transfer function (DTF). The remaining studies either employed or developed functional connectivity measures for their analysis. Weighted phase lag index (wPLI) and phase lag index (PLI) are much more frequently in use than any of the other functional connectivity measures. In Spyrou et al. (2019), complex tensor factorization (CTF) was proposed to estimate functional brain connectivity with EEG and in Mammone et al. (2018b), permutation Jaccard distance (PJD) is introduced to compute coupling strength between EEG time series. In Zhao et al. (2019), revised orthogonal least squares (ROLS) algorithm was proposed to measure connectivity between pairs of EEG signals, and in Song et al. (2019), a strategy was developed to evaluate the brain functional network based on generalized composite multiscale entropy vector (GCMSEV).

### EEG and MEG channel densities and epoch length of surveyed articles

Within the frame of the considered articles, it is found that all the seven articles that employed MEG signals used 306 channels, while 27.27% of the remaining 44 articles that employed EEG signals used HD-EEG montage and the rest used LD-EEG montage. Meanwhile, the largest proportion (29.54%) of the reviewed articles employed the use of 19 electrodes with no clear reason for the selection except for analysis convenience and the cost of equipment acquisition.

Similarly, with regards to the selection of the epoch length of the signals, it is found that there has been no specific consideration for the epoch length selection for the connectivity analysis of dementia-related disorders among the reviewed articles. The various channel densities and epoch lengths employed for the surveyed articles are presented in Table 4 for reference.

**Table 4:** Data type, channel density, epoch length and primary findings of the reviewed studies.

**S/N**	**Data type**	**Channel density**	**Epoch length (s)**	**Primary findings**	**References**
1	EEG	21	8.2	Selective involvement of frontal network in bvFTD against global efficiency and parietal and occipital loss of network organization in AD	Yu et al., 2016
2	HD-EEG	128	1	Presence of significantly higher local connectivity in AD than healthy controls in alpha and beta bands due to compensatory increase in local connectivity as a result of wide-spread decline in the long-range connections	Afshari and Jalili, 2016
3	HD-EEG	128	1	Detection of dependence of AD-related abnormalities on the methods employed for network dependency estimation and binarization	Jalili, 2016
4	EEG	16	8	Better discrimination of AD from NC based on the brain functional network constructed by limited penetrable visibility graph and phase space method than the analysis of single series, which is instrumental for revealing the underlying pathology the disease	Wang J. et al., 2016
5	EEG	19	2	Representation of neurophysiological biomarker of AD using functional connectivity disruptions between certain brain regions, as measured with lagged phase synchronization	Hata et al., 2016
6	EEG	32	0.6	Detection of a weakened outgoing information flow, a decrease in out-degree, and an increase in in-degree at the parietal region in VaD patients, compared to healthy controls	Wang C. et al., 2016
7	EEG	19	2	Representation of structural hippocampal atrophy functionally using small world connectivity pattern	Vecchio et al., 2017
8	MEG	306	10	Information flow hindrance between brain regions, particularly from posterior hub regions due to AD pathology, and indication of pathophysiological process of the disease in the information flow in beta band	Engels et al., 2017
9	HD-EEG	128	1	Stability of network properties across all frequency bands and also significant reduction of local efficiency and modularity measures in AD brains at eyes-closed condition	Jalili, 2017
10	MEG	306	16	Identification of increasing and decreasing PLI values in lower and upper alpha bands respectively in MCI patients which are interpreted as a dual pattern of disconnection and aberrant functioning	López et al., 2017
11	EEG	16	8	Discovery of a more homogeneous functional brain network in AD subjects and also a decline in small world efficiency of AD networks	Yu et al., 2018
12	EEG	19	2	Discovery of a more compromised neurophysiological reserve in ADD than DLB, at both group and individual levels using functional cortical connectivity markers in delta and alpha sources	Babiloni et al., 2018
13	HD-EEG	256	1	Formulation of compression strategy for HD-EEG signal reconstruction with minimum information loss in dementia related disorders	Mammone et al., 2018a
14	EEG	19	5	Objective evaluation of the connectivity density modifications associated to the MCI-AD conversion by mixing nonlinear analysis with a machine learning approach	Mammone et al., 2018b
15	EEG	16	8	Identification of elevated small world properties in the cross-frequency networks in AD compared to control, indicating an impaired balance between within and cross frequency interaction	Cai et al., 2018
16	EEG	16	8	Development of a novel EEG-based strategy for functional connectivity quantification and enrichment of topographical biomarkers used for neurophysiological assessment	Song et al., 2019
17	HD-EEG	128	2	Identification of brain connectivity and location of couples sources for EEG-based MCI dataset using approach that relies on tensor factorization	Spyrou et al., 2019
18	MEG	306	4	Development of dynamic network multi-frequency analysis approach for effective construction of a sensitive MEG-based connectome biomarker for the prediction of conversion from MCI to Alzheimer's disease	Pusil et al., 2019
19	EEG	19	2	Identification of abnormal lower widespread interhemispheric and intrahemispheric LLC solutions in alpha sources in both MCI groups compared with the NC group	Babiloni et al., 2019
20	EEG	19	16	Alteration of topological properties of network in AD patients also in its prodromal stage, starting with the reduction of edge density and then loss of the local and global efficiency	Franciotti et al., 2019
21	EEG	32	7	Characterization of AD-induced brain networks by lower degree, clustering coefficient at the frontal pole and medial orbitofrontal across all frequency range, compared to the networks of age-matched healthy controls	Li et al., 2019
22	HD-EEG	256	1	Identification of consistently weak small world properties of brain functional networks of MCI and AD patients formulated from HD-EEG compared to healthy subjects	La Foresta et al., 2019
23	HD-EEG	128	4	Proposition of a novel brain functional connectivity imaging technology aiming to determine the contribution of non-linearity and dynamics for AD and NC participants discrimination	Zhao et al., 2019
24	MEG	306	1	Adaptation of a novel data-driven thresholding scheme based on OMSTs and extraction of prototypical network microstates (FCμstates) for both the control and MCI group	Dimitriadis et al., 2019
25	EEG	19	9	Involvement of topological reorganization of brain functional network in the evolution of AD and importance of Network measures for the evaluation of symptom severity in AD	Chen et al., 2019
26	EEG	16	8	Discovery of the local efficiency and clustering coefficient as one of the most effective factors in AD identification at functional network level	Yu et al., 2020
27	EEG	30	2	Direct comparison of EEG and sMRI for differential identification of AD and aMCI	Farina et al., 2020
28	EEG	21	8	Improvement of functional connectivity in early AD, measured with AEC-c in the alpha frequency band upon treatment with PQ912	Briels et al., 2020a
29	EEG	32	5	Disintegration of functional network in alpha band under eyes open protocol and elevated hub strength in central region during cognitive task for the detection of early onset of AD	Das and Puthankattil, 2020
30	HD-EEG	64	2	Identification of significant decrease in functional connectivity and a less integrated graph topology MCI based networks ad also development of MCI prediction framework using a combination of functional connectivity, topological and cognition measurements	Požar et al., 2020
31	EEG	16	50	Application of multiplex framework to explore functional integration and segregation of brain networks and characterize the abnormalities of brain function	Cai et al., 2020
32	EEG	19	10	Formulation of extra discriminating information for MCI and NC by combining the weighted connectivity metrics	Jamaloo et al., 2020
33	EEG	21	10	Explanation of the atypical pattern of neurodegeneration in PCA-AD based on regional vulnerability of the posterior network	Briels et al., 2020b
34	EEG	19	20	The main frequency bands that are different between MCI patients and controls are the theta and lower alpha bands, and the affected brain areas are the frontal, left temporal and parietal areas	Duan et al., 2020
35	HD-EEG	128	2	Consistency of weighted network measures across global thresholding levels, and reflection of the network properties reduction of connectivity strength in the dementia groups	Mehraram et al., 2020
36	EEG	30	20	Effective analysis of brain networks of dementia disorder stages using statistical entropy	Abazid et al., 2021
37	EEG	19	2	Development of biomarkers that integrate connectivity, spectral characteristics, complexity and P300 diagnosing AD, MCI and NC	Toural et al., 2021
38	MEG	306	2	Prediction of the progression of patients with mild cognitive impairment (MCI) to AD, and identify brain regions with network alterations related to MCI	Xu et al., 2021
39	HD-EEG	256	1	Revelation of a higher robustness in the brain networks of healthy people, followed by MCI and, finally, by AD patients, consistent with the hallmarks of Alzheimer's disease based on graph measures	Dattola et al., 2021
40	EEG	16	1	Demonstration of the strength of directed brain network models for better classification for aMCI diagnosis compared to undirected networks	Li et al., 2021
41	HD-EEG	64	4	Discovery of a more prominent post-task resting state alteration of functional network in aMCI patients which could be employed as possible biomarkers of the disease	Youssef et al., 2021
42	EEG	18	1	Compared to the HC and PD groups, the PDMCI group is characterized by a more posterior topography of the delta-theta PAC and a reversed delta-low frequency alpha PAC direction	Bayraktaroǧlu et al., 2022
43	MEG	306	3.2	Resting-state functional connectivity changes in frontal, limbic and subcortical regions are accentuated in early symptomatic ALS patients and they overlap considerably with bvFTD	Govaarts et al., 2022
44	EEG	19	2	Lost of the main hub of HC (Parietal area) in FTD patients at onset of dementia, substituted by provincial hubs in frontal leads and no changes in global network organization in AD	Franciotti et al., 2022
45	EEG	19	2	Higher modularity is found in the beta band and lower radius in the gamma band in aMCI compared to naMCI	Kim et al., 2022
46	EEG	64	10	Functional connectivity improvement of the right PCC to the right DC is a possible mechanism by which overall cognitive and memory function in MCI patients improves through rTMS	Zhang et al., 2022
47	EEG	bi-polar 23	12	Development of GNN models to compare the performance of selected FC measures and for classification of AD and NC networks	Klepl et al., 2022
48	EEG	19	3.2	Detection of a decrease in the characteristic path lengths of the alpha1 band in the right supramarginal gyrus and right rostral middle frontal cortex were observed in participants who received intervention	Park et al., 2022
49	MEG	306	3.2	Demonstration of the potential for MEG biomarkers to increase diagnostic accuracy of cognitive decline and dementia in a noninvasive manner	Scheijbeler et al., 2022
50	EEG	30	8	Identification of a more severe pathological changes in EOAD patients compared to LOAD based on evaluation of coherence alterations and diagnostic value of coherence measure	Fide et al., 2022
51	EEG	30	20	Detection of a network that is more resilient to neuronal damage in SCI compared to that of MCI and even more compared to that of AD	Abazid et al., 2022

### Assessment of studies qualities

**Figure 5:**
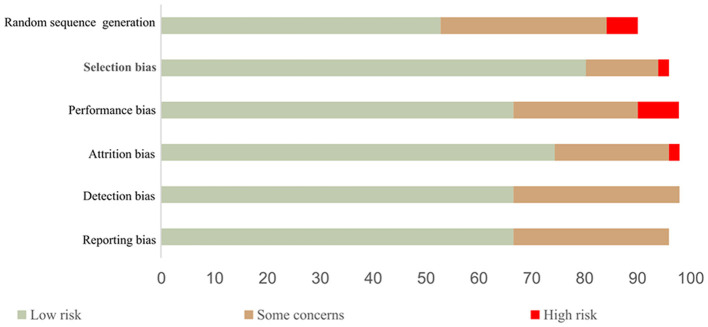
Risk of bias assessment using the Cochrane Collaboration tool.

The strength of the study's evidence was evaluated using the Cochrane Guidelines for Systematic Review of Diagnostic Test Accuracy (Smetana et al., 2012). The evaluated domains include random sequence generation, selection bias, performance bias, attrition bias, detection bias, and reporting bias, respectively. Evaluating the quality of evidence across studies, the lack of completeness, and missing data from the included studies was carefully examined. Thus, studies were categorized into three different groups (high, average, and low with a respective number of low-risk domain ≥4, = 3, ≤ 2), where high-quality studies were judged to have low bias, average-quality studies were judged to have unclear criteria and low-quality studies were judged to have a high bias. Out of the total of 51 studies considered, 34 are classed as high quality, 14 are classed as average quality, and three are classed as low quality as shown in Figure 5.

**Figure 6:**
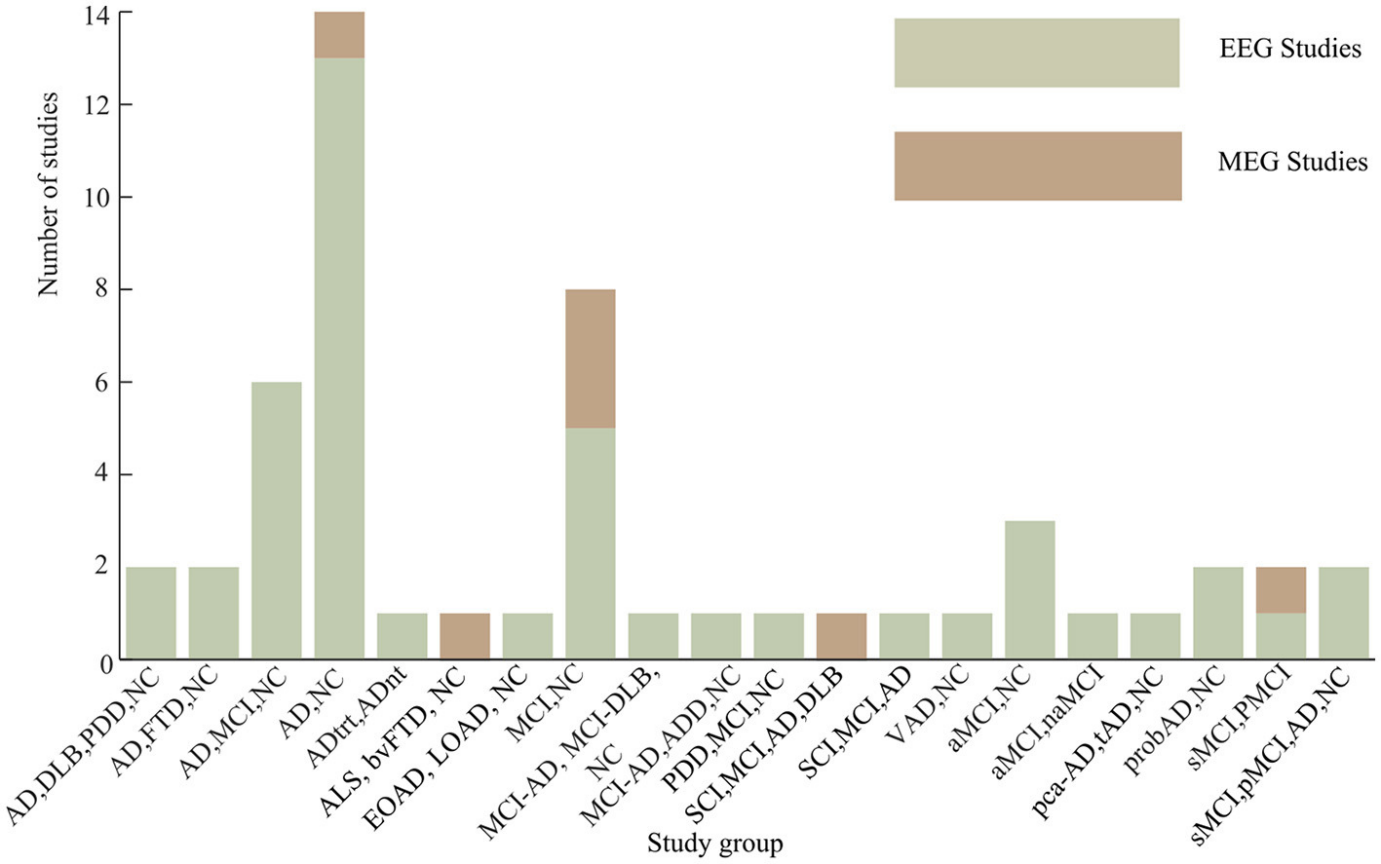
Groups of dementia-related disorders studied.

Among all the 51 articles considered, 14 articles (27.5% of the total studied articles) analyzed {AD, NC} groups. A total number of eight studies (15.7% of the total studied articles) was considered {AD, MCI, NC}, while a total of six articles (11.8% of the total studied articles) analyzed {MCI, NC} group. Other considered groups of dementia disorders are as presented in Figure 6. Stable mild cognitive impairment (sMCI) and progressive mild cognitive impairment (pMCI) groups were studied in three study articles. In Briels et al. (2020a), the efficacy of an inhibitor of the glutaminyl cyclase enzyme (PQ912) was evaluated in patients with early AD using functional connectivity. Similarly, Park et al. (2022) studied the neurophysiological changes in QEEG after 24-week multidomain lifestyle intervention program for the prevention of cognitive impairment in at-risk older adult individuals.

### Graph theory metrics and minimum spanning tree metrics

**Figure 7:**
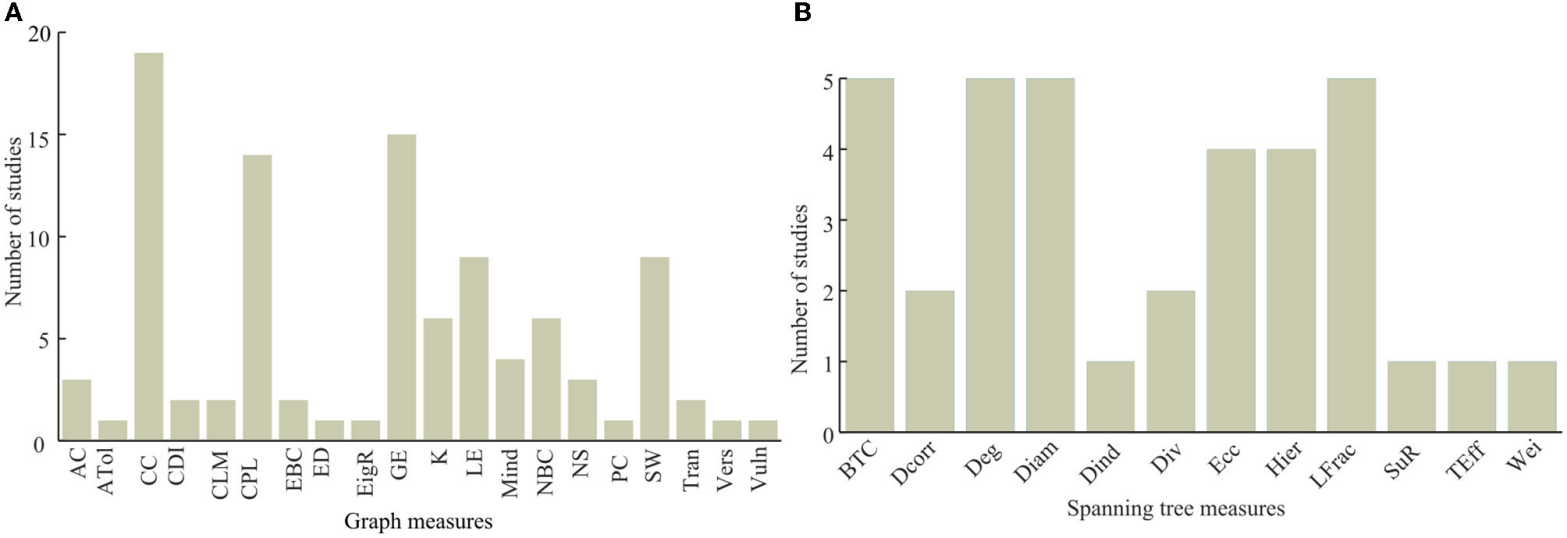
Usage of graph theory measures with **(A)** being the usage of graph theory metrics and **(B)** being the usage of spanning tree metrics.

As shown in Figure 7A, clustering coefficient (CC), characteristic path length (CPL), and global efficiency are the most frequently employed graph theory measures. Clustering coefficient has been used by 19 articles, while global efficiency and characteristic path length have been used by 15 and 14 articles, respectively. Small worldness (SW), local efficiency, node betweeness centrality, and nodal degree have also been frequently used as compared to the other metrics (see Figure 7). Similarly, the frequently used minimum spanning tree network metrics include the tree betweenness centrality (BTW), tree degree, tree diameter, and leaf fraction all of which have been used in five of the studied articles. Eccentricity (Ecc) and tree hierarchy have been employed for spanning tree network quantification in four articles each as presented in Figure 7B.

## Discussion

A lot of interesting findings regarding dementia-related disorders have unfolded within the last 5 years as a result of connectivity analysis of electrophysiology signals. Research into connectivity and brain network analysis of dementia disorders has gained attention and it is expected to attract more interest in the next couple of years as the rate of people living with dementia is constantly on the rise. Currently, about 55 million people or more live with dementia worldwide with about 60% of this population from the low- and middle-income nations of the world (WHO, 2021). This is one of the major reasons why electrophysiology signals especially EEG becomes a very important tool in the detection of the early onset of dementia and also in the differential diagnosis of the later stages. On account of the increase in the proportion of the older population in every country of the world, about 78 million people are projected to develop dementia in 2030 and about 139 million in 2050 (WHO, 2021). The expected rise of interest to research in this domain necessitates the review. Upon rigorous screening process, 51 pair review articles that satisfactorily meet the study criteria are systematically reviewed, and the summary of the primary findings of the included articles is presented in Table 4.

Overall, the percentage of the reviewed articles that focus on the use of MEG as electrophysiology signals for their analysis is about 14%, while the percentage of studies that used EEG for their analysis is about 86%. This could be attributed to the relative availability of EEG signals compared to MEG signals based on their installation cost and other logistics (Singh, 2014). Similarly, most of the studies considered concentrated more on the identification of AD and MCI groups despite the fact that other groups of dementia disorders are on this rise too. This is due to the fact that AD accounts for at least two times the proportion of other dementia disorders put together (WHO, 2021). Setting aside the focus on AD, a notable fraction of the reviewed studies also paid attention to the detection of the early onset of dementia (MCI). It is believed that the inability to develop a therapeutic treatment for dementia until now is the irreversible mechanism behind its formation, and so a lot of researchers have proposed the concept of addressing the impairment from the MCI stage. However, it is very difficult to discriminate between normal aging and the early/MCI stage of dementia. This is the reason why there is an increasing interest toward differential diagnosis between normal aging and MCI stages. One of the reviewed studies focused on evaluating the efficacy of an anti-dementia medicine (glutaminyl cyclase enzyme (PQ912), and found increasing functional connectivity in alpha frequency band after the treatment of patients with early dementia with PQ912 (Briels et al., 2020a). However, further validation of the study over a large sample population is necessary. Furthermore, it is found that the majority of the reviewed articles focus on AD as the most prevalent form of dementia disorders. However, it is not a new concept that most dementia disorders have overlapping clinical symptoms and so it becomes necessary for experts in this domain to look into developing discriminatory frameworks for the differential diagnosis of dementia-related disorders.

Going by the trend of dementia-related research and the development of therapeutic measures, the most promising direction is the early identification of the patients at risk of developing dementia in future. This discrimination possibility will ensure secondary prevention as a solution to the seemingly irreversible disorders (Weintraub et al., 2018). However, the present study shows that the functional and effective connectivity-based analysis of electrophysiology signals is lacking in this direction. From the 41 articles reviewed, only two articles (one for each of EEG and MEG) focused on the identification of the stable from the progressive mild cognitive impairment (see Figure 6).

With regard to the connectivity analysis of dementia-related disorders, the reviewed studies employed the use of different connectivity types for their analysis. The majority of the studies employed functional connectivity tools (about 85% of the total connectivity usage), while effective connectivity tools were employed in about 15% of the total connectivity usage. As there are different metrics used for accessing functional connectivity between pairs of electrodes/pair of region of interest, most of the study's articles employed those metrics that measure functional connectivity using the phase information of the signals. This may be attributed to the fact that the phase information of electrophysiology signals is more sensitive to neurological states than amplitude information. It is very important to be aware of the strengths and weaknesses of all forms of functional and effective connectivity measures. Some measures are highly sensitive to volume conduction, while some are quite robust. The linearity of a measure as well as the domain of measures are all ingredients to be aware of before deciding the employment of a particular measure for analysis. Table 2 provides detailed information about the properties of the frequently used measures within the last 5 years to aid easy access.

As the conversion of the connectivity matrix to the brain network is very crucial in brain network analysis of dementia disorders, threshold selection, a tool used for connectivity-network transformation plays a vital role in the analysis process. Threshold selection tends to have a significant influence on the results of the complete analysis. Ever since, the vast body of literature in this domain has shown that there has been a dichotomy with respect to threshold selection, with no golden rule absolutely accepting a method. Very recently, data-driven approaches have been proposed and they appear to have advantages over the previously common arbitrary/random selection. The important criteria that should be fundamental to any data-driven approach are the maintenance of connectedness, formation of comparable network densities across a group of networks in the same analysis, and formation of network with small-world properties among others. Unfortunately, no single known approach has considered these approaches in unison, and this is part of the reason why approaches such as MST and MCC are gaining unprecedented attention in the general brain network mapping of electrophysiology signals using functional connectivity. On this note, the development of a threshold selection framework that considers the aforementioned criteria is desired. Looking into threshold selection development on the basis of the eigenvalues of connectivity matrices might be very interesting in this regard.

For the automatic classification of dementia-related disorders, various traditional machine learning-based classifiers such as the support vector machine (SVM), random forest, linear discriminant analysis, and k-nearest neighbors. have been previously employed to classify or recognize various neurological states based on electrophysiological signals. In the last one decade, deep learning has been very popular and has found various applications in different domains with its superb power of performing automatic feature extraction and prediction or classification. One of the frequently used deep learning techniques for functional/effective brain network classification is the graph convolution network (GCN). GCN has been successfully applied for emotion prediction, brain-computer interface, and other applications (Lun et al., 2020; Zhong et al., 2020; Chen et al., 2021). However, the advantage of various deep learning techniques including GCN is underutilized in electrophysiology-based brain network analysis of dementia-related disorder. It is, therefore, recommended that GCN, transformer network, and other deep learning techniques should be employed for the automatic discrimination of these disorders with overlapping underlying mechanisms.

## Conclusion

This article features an increasing trend in the studies of electrophysiology signal-based functional and effective brain networks of dementia-related disorders over the last 5 years. The article mainly focuses on two electrophysiology signals; EEG and MEG especially because of their high temporal resolutions and also because of the acquisition cost (for EEG) which makes these modalities serve as promising tools for the diagnosis of dementia disorders. Most of the articles reviewed employed electrophysiology data at rest (eyes-open or eyes-closed). Although it may be difficult to record data from dementia-related subjects during cognitive task protocol, designing and achieving such protocols could improve our understanding of the underlying mechanism of dementia-related brain networks. The various measures of connectivity that have been used up to date are highlighted with their respective properties to guide the usage selection. The conversion of the connectivity matrix into brain networks using the threshold selection approach is also discussed together with details of the very recently proposed and employed techniques. Recommendations are made to the important criteria that can enable the development of unbiased threshold selection.

The emergence of graph theory metrics as valuable ingredients of functional and causal brain network analysis of dementia-related disorders has also been reviewed. It is found that clustering coefficient (CC), global efficiency, and characteristic path length (CLP) are more frequently employed in the analysis of dementia-related disorders. Various machine learning techniques recently employed for the classification of dementia-related disorders based on functional/causal brain network features are also identified. As deep learning techniques have been underutilized in this domain, recommendations are made to harness its full potential for dementia-related identification basic on the complex network theory framework.

## Data availability statement

The original contributions presented in the study are included in the article/supplementary material, further inquiries can be directed to the corresponding author.

## Author contributions

AA: study design, data collection and analysis, drafting and revision of the manuscript, and figure preparation. KV: conceptualization, study design, and revision of the manuscript. All authors contributed to the article and approved the submitted version.
